# SUDEP and mortality in developmental and epileptic encephalopathies: A meta‐analysis of randomized clinical trials and extension studies

**DOI:** 10.1002/epi.70348

**Published:** 2026-06-26

**Authors:** Pierludovico Moro, Maria Sole Borioni, Adolfo Mazzeo, Enrico Cocchi, Carlo Di Bonaventura, Emanuele Cerulli Irelli

**Affiliations:** ^1^ Department of Human Neurosciences Sapienza University Rome Italy; ^2^ Department of Medical and Surgical Sciences University of Bologna Bologna Italy

**Keywords:** childhood, Dravet syndrome, epilepsy, infantile epileptic spasm syndrome, Lennox–Gastaut syndrome

## Abstract

**Objective:**

Developmental and epileptic encephalopathies (DEEs) are associated with high premature mortality and increased risk of sudden unexpected death in epilepsy (SUDEP). However, epidemiological data remain limited, particularly for specific syndromes such as Dravet syndrome (DS), Lennox–Gastaut syndrome (LGS), and infantile epileptic spasms syndrome (IESS). To address this gap, we conducted a Bayesian meta‐analysis of randomized controlled trials (RCTs) and corresponding long‐term open‐label extension (OLE) studies.

**Methods:**

All‐cause mortality and SUDEP incidence rates were estimated using hierarchical Bayesian models with moderately informative priors derived from the literature. A negative binomial likelihood was used for all‐cause mortality, whereas a Poisson likelihood was used for SUDEP. Subgroup analysis were conducted for DS, LGS, and IESS.

**Results:**

Thirty‐seven RCTs and 15 OLE studies were included, comprising 3757 patients with DEEs (2221 LGS, 998 DS, 369 IESS, and 169 other DEEs). Across pooled data, 37 deaths occurred, including 2 definite, 12 probable, and 1 possible SUDEP. In the overall DEE population, the Bayesian posterior median estimated mortality rate was 8.76 per 1000 person‐years (PY; 95% credible interval [CrI], 5.50–13.89), and SUDEP rate was 4.32 per 1000 PY (95% CrI, 2.66–6.64). In LGS, mortality was 7.05 per 1000 PY and SUDEP was 3.4 per 1000 PY. In DS, mortality was 8.0 per 1000 PY, with a SUDEP rate of 7.59 per 1000 PY. In IESS, mortality was 9.41 per 1000 PY and SUDEP was 2.79 per 1000 PY.

**Significance:**

SUDEP accounted for approximately half of deaths in the overall DEE population and in LGS, with a proportionally greater contribution in DS. Potential trial‐related underestimation should be considered when interpreting these findings, as features of trial‐based datasets could have contributed to lower observed mortality and SUDEP estimates. Prospective population‐based studies are needed to more accurately define the real‐world incidence of SUDEP in DEEs.

**Key points**

- Overall sudden unexpected death in epilepsy (SUDEP) rate was ~4.3 per 1000 person‐years (PY); mortality was ~8.8 per 1000 PY in developmental and epileptic encephalopathy (DEE) trial populations.
- SUDEP rates were highest in Dravet syndrome (DS); Lennox–Gastaut syndrome (LGS) and infantile epileptic spasms syndrome (IESS) showed lower but still substantial estimates.
- Trial‐based data may underestimate SUDEP and mortality due to short follow‐up and selection bias.
- Findings highlight SUDEP as a major cause of death and the need for real‐world long‐term data in DEEs.

## 1 INTRODUCTION

Developmental and epileptic encephalopathies (DEEs) are a heterogeneous group of severe epilepsy syndromes characterized by drug‐resistant seizures and developmental impairment or regression, in which both the underlying developmental abnormality and the epileptic activity itself contribute to cognitive, behavioral, and neurological dysfunction. In DEEs, developmental slowing or regression may result from the underlying etiology, recurrent seizures, interictal epileptiform activity, or a combination of these factors, although the relative contribution of each component is often difficult to disentangle.^1^, ^2^

DEEs are associated with substantial premature mortality, including an increased risk of sudden unexpected death in epilepsy (SUDEP), defined as a sudden, unexpected, witnessed or unwitnessed, non‐traumatic, and non‐drowning death in an individual with epilepsy, occurring under benign circumstances, with or without evidence of a preceding seizure, and excluding deaths due to documented status epilepticus or those in which post‐mortem examination reveals an alternative cause.^3^, ^4^

A recent retrospective cohort study of individuals with DEEs estimated an overall mortality rate of 6.1 per 1000 person‐years (PY) (95% confidence interval [CI] 4.4–8.3) and a SUDEP rate of 2.8 per 1000 PY (95% CI 1.6–4.3), with a significant association observed for pathogenic variants in genes such as *SCN1A*, *SCN2A*, *SCN8A*, and *STXBP1*.^4^ Recent reviews further indicate that SUDEP incidence varies across DEE subgroups, with Dravet syndrome (DS) associated with the highest mortality, with SUDEP reported as the most frequent cause of death (estimated incidence of 15.84 per 1000 PY), markedly higher than that observed in other pediatric epilepsies. In Lennox–Gastaut syndrome (LGS), the estimated mortality incidence is 6.12 per 1000 PY, and, to our knowledge, no systematic data on SUDEP incidence are currently available for this population.^5^, ^6^, ^7^, ^8^ In infantile epileptic spasms syndrome (IESS), SUDEP appears to be rare, with most deaths attributed to the underlying etiology or medical complications rather than SUDEP; however, published studies do not provide precise SUDEP incidence estimates for this subgroup.^8^, ^9^, ^10^, ^11^ Overall, evidence from large, prospective DEE cohorts with regard to SUDEP is limited, and comprehensive global epidemiological data are lacking, especially for DS, LGS, and IESS.

Despite major advances in the therapeutic landscape, this paucity of data continues to hinder a clear understanding of diagnostic trajectories and mortality risk for individuals with DEEs. Over the past decades, multiple clinical trials have been designed to target DEEs broadly, specific DEE syndromes, or genetically defined DEEs.^12^ SUDEP rate estimates derived from clinical trials are often constrained by small sample sizes, limited follow‐up durations, and the rarity of SUDEP events, thereby limiting the precision and generalizability of these estimates. However, they offer standardized outcome ascertainment with mandatory adverse event (AE) reporting and prospectively monitored exposure time, which may reduce misclassification bias. Notably, no previous study has provided pooled, incidence‐based SUDEP estimates derived from prospectively monitored clinical trial populations in DEEs.

Therefore, to provide more robust incidence estimates in a large‐scale population, we conducted a Bayesian meta‐analysis of randomized controlled trials (RCTs) and corresponding long‐term open‐label extension (OLE) studies including patients with DEEs, with the aim of estimating mortality and SUDEP rates both in the overall DEE population and across clinically relevant subgroups such as LGS, DS, and IESS.

## 2 METHODS

### 2.1 Standard protocol approvals and registrations

This meta‐analysis was registered in the International Prospective Register of Systematic Reviews (PROSPERO) (ID: CRD420251267429) and conducted in accordance with the Preferred Reporting Items for Systematic reviews and Meta‐Analysis (PRISMA) guidelines,^13^ as detailed in the Data S1 (eTable 1).

### 2.2 Data Collection and Synthesis

Systematic literature searches were conducted in PubMed, the Cochrane Library, and the ClinicalTrials.gov registry from database inception through December 18, 2025. In addition, reference lists of relevant reviews and included studies were screened manually to identify further eligible trials. The full search strategy is provided in the Supporting Information.

When detailed information on deaths was not available in published articles, study sponsors were contacted; however, no further data could be obtained. Supplementary searches were also performed using U.S. Food and Drug Administration (FDA) clinical reviews associated with original drug approvals.^14^

### 2.3 Eligibility criteria and screening

We included peer‐reviewed, English‐language RCTs. Study records with reported results published in ClinicalTrials.gov and reviewed by the National Library of Medicine were also eligible. The study population comprised patients of any age diagnosed with DEEs enrolled in pharmacological RCTs evaluating anti‐seizure medications (ASMs). All records identified through the search strategy were uploaded into Rayyan,^15^ and duplicates were removed. Two reviewers (P.M. and M.S.B.) independently screened titles and abstracts and categorized records as “excluded,” “included,” or “maybe,” with the latter two categories proceeding to full‐text review. Full texts were independently assessed by the same reviewers. Disagreements were resolved through discussion with a third reviewer (E.C.I.). Studies meeting inclusion criteria were retained for data extraction and analysis.

### 2.4 Data extraction

Data extraction was performed independently by two reviewers (P.M. and M.S.B.) using a standardized extraction form. Extracted variables included study characteristics, participant characteristics, and reported outcomes (Table 1). For each eligible RCT, linked publications, trial registry records (ClinicalTrials.gov), and cited references were screened to identify corresponding long‐term OLE studies reporting extended follow‐up of the same study population. Trial identifiers and sponsor information were used to confirm linkage between RCTs and their respective OLE phases. Only OLE studies directly derived from included RCTs and reporting mortality or SUDEP outcomes were eligible. This structured approach ensured traceability while preserving the systematic nature of the review.

**TABLE 1:** Characteristics of the included studies and reported mortality events.

RCT (author/ID, year)	ASM	Control group	OLE phase? (yes/No)	Patients (*n* RCT, OLE)	Age group (adult, pediatric, mixed)	Mean follow‐up duration, weeks (RCT, OLE)	Epileptic syndrome	Deaths (*n* RCT, OLE)	SUDEP (*n* RCT, OLE)
Devinsky, 2021	Ataluren	Placebo	No	15, −	Pediatric	12, −	DS and CDKL5‐DEE	0, −	0, −
Dlugos, 2025	Bexicaserin	Placebo	No	52, −	Mixed	10, −	LGS	0, −	0, −
Devinsky, 2018 (GWPCARE1a)	Cannabidiol	Placebo	Yes ^[^ ^16^ ^]^	34, 264	Pediatric	10, 36.4	DS	0, 2	0, 2
Devinsky, 2017 (GWPCARE1b)	Cannabidiol	Placebo	Yes ^[^ ^16^ ^]^	120, 264	Pediatric	14, 36.4	DS	0, 2	0, 2
Miller, 2020 (GWPCARE2)	Cannabidiol	Placebo	Yes ^[^ ^16^ ^]^	199, 264	Pediatric	14, 36.4	DS	0, 2	0, 2
Devinsky, 2018 (GWPCARE3)	Cannabidiol	Placebo	Yes ^[^ ^17^ ^]^	225, 366	Mixed	14, 19.2	LGS	0, 11	0, 3
Thiele, 2018 (GWPCARE4)	Cannabidiol	Placebo	Yes ^[^ ^17^ ^]^	171, 366	Mixed	14, 19.2	LGS	1, 11	0, 3
Ng, 2011	Clobazam	Placebo	Yes ^[^ ^18^ ^]^	238, 267	Mixed	15, 104	LGS	0, 10	0, 4
NCT00162981, 2012	Clobazam	Clobazam at different dose (low vs. high dose)	Yes ^[^ ^18^ ^]^	68, 267	Mixed	4, 104	LGS	0, 10	0, 4
NCT04639310, 2020	Ezogabine	Placebo	No	8, −	Pediatric	16, −	KCNQ2‐DEE	0, −	0, −
Ritter, 1993	Felbamate	Placebo	Yes ^[^ ^19^ ^]^	73, 70	Mixed	9, 48	LGS	0, 0	0, 0
Siegel, 1999	Felbamate	Placebo	No	13, −	Pediatric	7, −	LGS	0, −	0, −
Nabbout, 2019	Fenfluramine	Placebo	Yes ^[^ ^20^ ^]^	87, 232	Mixed	15, 34.8	DS	1, 0	1, 0
Sullivan, 2023	Fenfluramine	Placebo	Yes ^[^ ^20^ ^]^	143, 232	Pediatric	14, 34.8	DS	1, 0	1, 0
Lagae, 2019	Fenfluramine	Placebo	Yes ^[^ ^20^ ^]^	119, 232	Pediatric	14, 34.8	DS	0, 0	0, 0
Knupp, 2022	Fenfluramine	Placebo	Yes ^[^ ^21^ ^]^	263, 247	Mixed	14, 48.4	LGS	1, 1	1, 0
Knight, 2022	Ganaxolone	Placebo	Yes ^[^ ^22^ ^]^	101, 88	Pediatric	17, 96	CDKL5‐DEE	0, 1	0, 0
Sullivan, 2023	Ganaxolone	Placebo	No	21, −	Pediatric	17, −	PCDH19‐DEE	0, −	0, −
NCT00441896, 2007	Ganaxolone	Placebo	No	57, −	Pediatric	17, −	IESS	0, −	0, −
Motte, 1997	Lamotrigine	Placebo	No	169, −	Mixed	16, −	LGS	0, −	0, −
NCT04625101, 2020	NBI‐827104	Placebo	No	24, −	Pediatric	13, −	DEE‐SWAS	0, −	0, −
Vossler, 2024	Perampanel	Placebo	Yes ^[^ ^23^ ^]^	71, 58	Mixed	18, 46	LGS	0, 2	0, 1
Arzimanoglou, 2019	Rufinamide	Any other investigator‐chosen ASM	No	37, −	Pediatric	112, −	LGS	1, −	0, −
Glauser, 2008	Rufinamide	Placebo	Yes ^[^ ^24^ ^]^	138, 124	Mixed	12, 59.6	LGS	0, 0	0, 0
Ohtsuka, 2014	Rufinamide	Placebo	Yes ^[^ ^25^ ^]^	59, 54	Mixed	12, 102.4	LGS	0, 0	0, 0
Hahn, 2022	Soticlestat	Placebo	No	139, −	Pediatric	20, −	DS and LGS	0, −	0, −
Halford, 2021	Soticlestat	Placebo	Yes ^[^ ^26^ ^]^	18, 16	Adult	4, 7.2	LGS	0, 0	0, 0
NCT04938427, 2021	Soticlestat	Placebo	No	270, −	Mixed	16, −	LGS	0, −	0, −
NCT04940624, 2021	Soticlestat	Placebo	No	144, −	Mixed	16, −	DS	1, −	1, −
Chiron, 2000	Stiripentol	Placebo	No	42, −	Mixed	8, −	DS	0, −	0, −
Guerrini, 2002	Stiripentol	Placebo	No	23, −	Pediatric	NA	DS	0, −	0, −
Debus, 2004	Sulthiame	Placebo	Yes ^[^ ^27^ ^]^	37, 36	Pediatric	1, 64	IESS	0, 0	0, 0
Sachdeo, 1999	Topiramate	Placebo	Yes ^[^ ^28^ ^]^	98, 97	Mixed	3, 71.6	LGS	0, 0	0, 0
Striano, 2022	Triheptanoin	Placebo	Yes ^[^ ^29^ ^]^	36, 34	Mixed	10, 42	GLUT‐1 deficiency syndrome	0, 0	0, 0
Appleton, 1999	Vigabatrin	Placebo	Yes ^[^ ^30^ ^]^	40, 36	Pediatric	1, 24	IESS	0, 1	0, 0
Elterman, 2001	Vigabatrin	Vigabatrin at different dose (low vs. high dose)	Yes ^[^ ^31^ ^]^	227, 221	Pediatric	2, 156	IESS	2, 1	1, 0
Dyken, 1985	Valproic Acid	Placebo	No	8, −	Pediatric	17, −	IESS	0, −	0, −

Abbreviations: ASM, anti‐seizure medication; DEE, Developmental and Epileptic Encephalopathy; DEE‐SWAS, Developmental and Epileptic Encephalopathy with Spike–Wave Activation in Sleep; DS, Dravet syndrome; GLUT‐1, Glucose Transporter Type 1; ID, Identification; IESS, Infantile Epileptic Spasms Syndrome; LGS, Lennox–Gastaut syndrome; OLE, Open‐Label Extension; RCT, Randomized Controlled Trial; SUDEP, Sudden Unexpected Death in Epilepsy.

Two expert epileptologists (C.D.B. and E.C.I.) independently and blindly reviewed all available information for each reported death, including data from original publications, sponsors, and FDA clinical reviews (summarized in Table 2). When deaths were clearly classified as SUDEP in the original publication and no additional information was available, they were categorized as probable SUDEP in the present analysis. In addition, individual patient‐level information was searched in FDA clinical reviews and relevant publications to retrieve autopsy data for definite SUDEP cases (when available),^32^ identify possible SUDEP cases in individuals with competing causes of death, and review the reported cause of death for each included case.

**TABLE 2:** Deaths and SUDEP classification according to the information provided by the original trials or FDA documents.

Study name	Study phase	Study drug	Cause of death and details	SUDEP classification	Treatment arm (RCT phase)
Devinsky, 2019	OLE	Cannabidiol	SUDEP (considered unrelated to treatment)	Probable	Not applicable
Devinsky, 2019	OLE	Cannabidiol	SUDEP (considered unrelated to treatment)	Probable	Not applicable
Patel, 2021	OLE	Cannabidiol	9 deaths (2 SUDEP, 7 other causes)	Probable	Not applicable
Patel, 2021	OLE	Cannabidiol	Death due to convulsion (the death occurred during sleep, but the patient had fever, low oxygen saturation, and reduced urine output for 4 days and was seen in the emergency department the day before death)	Possible	Not applicable
Thiele, 2018	RCT	Cannabidiol	ARDS	No SUDEP	ASM
Conry, 2014	OLE	Clobazam	ARDS and right leg hematoma (died during a hospitalization for urosepsis, right leg hematoma with anemia, and valproate toxicity with thrombocytopenia)	No SUDEP	Not applicable
Conry, 2014	OLE	Clobazam	Pneumonia	No SUDEP	Not applicable
Conry, 2014	OLE	Clobazam	Pneumonia (respiratory insufficiency due to bilateral pneumonia that resulted from aspiration)	No SUDEP	Not applicable
Conry, 2014	OLE	Clobazam	Pneumonia	No SUDEP	Not applicable
Conry, 2014	OLE	Clobazam	Death of unknown origin or etiology (found dead in bed, at home without clear cause of death; in history, the patient had chronic lung disease and obstructive sleep apnea)	Probable	Not applicable
Conry, 2014	OLE	Clobazam	Death of unknown origin or etiology (found dead in bed during sleep, at home without clear cause of death; the reported cause of death was epilepsy)	Probable	Not applicable
Conry, 2014	OLE	Clobazam	SUDEP (cardiopulmonary arrest. The autopsy determined that the cause of death was “natural causes” with underlying seizure disorder related to perinatal asphyxia)	Definite	Not applicable
Conry, 2014	OLE	Clobazam	Pneumonia, sepsis, and ARDS (The subject experienced an extended seizure with aspiration and developed a fever and was subsequently hospitalized for pneumonia)	No SUDEP	Not applicable
Conry, 2014	OLE	Clobazam	Pneumonia and cardiopulmonary arrest	No SUDEP	Not applicable
Conry, 2014	OLE	Clobazam	Preexisting seizure increase, atelectasis, and hypoxic respiratory failure (the subject was hospitalized for convulsions and after clinical seizures ceased, EEG found ongoing subclinical seizures. The subject's hospital course was also notable for desaturation episodes. The subject had a salivagram that demonstrated aspiration. His G tube was converted to a GJ tube. He subsequently experienced respiratory failure and died)	No SUDEP	Not applicable
Sullivan, 2020	OLE	Fenfluramine	SUDEP (considered not related to study drug. Patient had died during sleep. The autopsy report listed the cause of death as “Seizure disorder, due to Dravet syndrome.” Other findings on autopsy were lung changes consistent with a focal infection, possibly pneumonia)	Definite plus	Not applicable
Sullivan, 2023	RCT	Fenfluramine	SUDEP (considered not related to study drug. The patient woke up, and said she felt tired and wanted to go back to bed. She returned to bed, and about 30 to 40 min later, the mother found the child face‐down in bed with her hands clenched, blue, and pale. No autopsy was performed)	Probable	Placebo
Knupp, 2022	RCT	Fenfluramine	SUDEP (considered not related to study drug. The patient e was found dead after taking a nap. No autopsy was performed)	Probable	ASM
Knupp, 2022	OLE	Fenfluramine	Severe convulsive status epilepticus and aspiration pneumonia (considered unrelated to study drug)	No SUDEP	Not applicable
Olson, 2023	OLE	Ganaxolone	Sepsis (considered unrelated to study drug)	No SUDEP	Not applicable
Vossler, 2024	OLE	Perampanel	SUDEP	Probable	Not applicable
Vossler, 2024	OLE	Perampanel	Unknown cause	Unclassified	Not applicable
Arzimanoglou, 2019	RCT	Rufinamide	Pneumonia (considered unrelated to the study drug)	No SUDEP	ASM
NCT04940624	RCT	Soticlestat	SUDEP	Probable	ASM
Appleton, 1999	OLE	Vigabatrin	Acute respiratory infection and a cardiac arrest (not considered to be related to the study drug. The patient suffered mild bronchitis and a cardiac arrest leading to death; this was considered to be related to a heart disorder, possibly related to a previous infection)	No SUDEP	Not applicable
Elterman, 2001	RCT	Vigabatrin	SUDEP (at 1:30 pm, the infant's caregiver put her down for a nap. When the caregiver attempted to wake the infant at 3:30 pm, the infant was not breathing. The child had no intercurrent illness. An autopsy was performed; however, repeated attempts to obtain the report were unsuccessful)	Probable	Not reported
Elterman, 2001	RCT	Vigabatrin	Pulmonary hemorrhage secondary to pulmonary angiomatosis (The infant was in hospice care and had developed increasing spasm activity the weekend prior to her death. At midnight on the day before her death, she began to bleed from the mouth and expired 2 h later. Autopsy findings showed a pulmonary hemorrhage secondary to pulmonary angiomatosis. Plexuses of large abnormal muscularized vessels with focal subintimal fibrosis were present next to the right and left bronchi of the lungs, which raises the possibility of arteriovenous shunting. Left ventricular hypertrophy in the heart is additional evidence that there was an abnormal hemodynamic state)	No SUDEP	ASM
Elterman, 2010	OLE	Vigabatrin	Pneumonia (The patient was hospitalized with diagnosis of upper lobe pneumonia. “Do Not Resuscitate” orders were written at least 24 h prior to her death. No autopsy was performed)	No SUDEP	Not applicable

Abbreviations: AE, Adverse Event; AEDs, Antiepileptic Drugs; ASM, Antiseizure medication; ARDS, Acute Respiratory Distress Syndrome; CBZ, Carbamazepine; CLB, Clobazam; CPR, Cardiopulmonary Resuscitation; CT, Computed Tomography; EEG, Electroencephalogram; EMS, Emergency Medical Services; FFA, Fenfluramine; GJ tube, Gastrojejunostomy Tube; G‐tube, Gastrostomy Tube; ICU, Intensive Care Unit; LGS, Lennox–Gastaut Syndrome; NA, Not Available; OLE, Open‐Label Extension; RCT, Randomized Controlled Trial; SUDEP, Sudden Unexpected Death in Epilepsy; VGB, Vigabatrin; VNS, Vagus Nerve Stimulation; VPA, Valproic Acid.

We extracted the number of deaths, cases of SUDEP and related information, and total exposure time expressed in PY for each included study. Because reporting standards differed between RCTs and OLEs, exposure was calculated according to study phase. In the double‐blind RCT phases, PY were estimated from the safety population and the planned study duration using the formula PY = *N* × Study Duration (weeks/52), where *N* is the number of participants. When available, treatment allocation (placebo vs ASM) at the time of death or SUDEP was extracted and reported.

For OLE phases, variable follow‐up durations and participant attrition necessitated a hierarchical estimation approach. When the mean follow‐up duration was reported, PY were calculated as the product of the total number of participants and the mean follow‐up. When only the total study duration and the number of completers were reported, a midpoint assumption was applied,^33^ whereby participants completing the study were assumed to contribute the full study duration, whereas those who discontinued contributed, on average, half of the study duration, giving PY estimated = (*N* completers × duration) + (*N* dropouts × 0.5 × duration).

Data from the RCT and OLE phases were pooled into a unified analytical dataset, with each phase treated as a separate observation. In most cases, a single RCT was followed by its corresponding OLE; however, in some instances, multiple independent RCTs contributed participants to the same extension cohort. To account for this structure, related trials and their corresponding extension phases were grouped under a shared clinical development program identifier, which was used as random intercept (see Statistical Analysis).

This pooled approach was adopted for several reasons. First, because death is an absorbing event, participants who died during the RCT phase did not contribute person‐time to the OLE phase, preventing double‐counting of events or exposure.^34^ Second, clustering at the program level accounted for shared methodological characteristics—such as eligibility criteria, investigator networks, adjudication procedures, and study drug—that could induce intra‐program correlation. Although OLE cohorts represent a survivor population, the very low mortality observed during the short RCT phases suggests minimal depletion of susceptible individuals. Finally, given the limited follow‐up duration of RCTs in DEEs, this approach increased total exposure time and improved the precision of mortality and SUDEP estimates.

### 2.5 Outcomes

The primary outcome was SUDEP, defined according to current standardized criteria.^3^ Reported deaths were classified as definite (met all SUDEP criteria with complete postmortem examination that did not reveal an alternative cause of death), definite plus (met the definite SUDEP criteria with a health condition or autopsy/toxicologic finding that could have contributed to death), probable (met the criteria for definite SUDEP without postmortem examination), probable plus (met the probable SUDEP criteria with a health condition that could have contributed to death), possible (competing cause of death), not SUDEP (a clear alternative cause of death is identified), or unclassified (incomplete information available). The primary analysis included definite, probable, and possible SUDEP cases, whereas secondary analysis were restricted to definite and probable cases. The secondary outcome was all‐cause mortality.

### 2.6 Statistical analysis

Incidence rates were estimated using a Bayesian hierarchical generalized linear mixed‐model (GLMM). To account for potential overdispersion, overall mortality was modeled using a negative binomial likelihood. In contrast, SUDEP was modeled using a Poisson likelihood, as the extreme rarity of events and posterior predictive checks did not indicate overdispersion (see Supporting Information for detailed information on model specification and priors). Between‐study heterogeneity was modeled using a random intercept assigned to the overall drug development program, to account for between‐program heterogeneity and within‐program correlation. PY of follow‐up were incorporated as an offset to model incidence rates rather than raw event counts.

Given the rarity of events and sparse counts, moderately informative priors were specified. For all‐cause mortality, the overall log‐incidence rate was assigned a Normal (−4.27, standard deviation ‐SD‐ 0.5) prior, informed by previously reported mortality rates (~14 events per 1000 PY) in patients with LGS, DS, and IESS, as well as by a recent study in patients with epilepsy (see Supporting Information for further details).^5^, ^6^, ^7^, ^8^, ^9^, ^10^, ^11^, ^35^ For SUDEP, the overall log‐incidence rate was assigned a Normal(−4.96, SD 0.5) prior, corresponding to ~7 events per 1000 PY, based on the same previously reported literature and assuming that SUDEP accounts for ~48% of all deaths, as reported in prior studies.^5^ Between‐study heterogeneity was modeled using a half‐normal prior with scale 0.5 on the log‐rate scale, allowing moderate heterogeneity (approximately ±65% multiplicative variation per SD) while constraining implausible overdispersion.

To evaluate the influence of prior assumptions, we conducted sensitivity analysis allowing weakly informative and flat priors for the baseline intercept (SD = 2 and SD = 10 on the log scale, respectively), as well as alternative specifications for the between‐study heterogeneity parameter. In addition, estimates were compared with those derived from a frequentist Poisson GLMM, using the same random‐effects structure as the Bayesian model, to assess consistency between approaches. A sensitivity analysis restricted to RCT and OLE phases was also performed to evaluate mortality and SUDEP estimates within each phase separately.

Posterior incidence rates per 1000 PY were obtained by exponentiating the posterior draws of the model intercept and scaling the resulting rates to 1000 PY. Point estimates were reported as posterior medians of the transformed rate distribution, with 95% credible intervals (CrIs) defined by the 2.5th and 97.5th percentiles. Additional subgroup analysis were performed for LGS, DS, and IESS to derive syndrome‐specific incidence estimates using priors informed by previously reported mortality rates (see Supporting Information). Given the smaller sample sizes expected in these subgroup analysis, weakly informative priors were used to stabilize the models while allowing the estimates to be primarily driven by the observed data.

Models were fitted using the brms package in the R programming language.

### 2.7 Meta‐regression

To explore potential study‐level moderators of all‐cause mortality and SUDEP, Bayesian mixed‐effects meta‐regressions were conducted. Mean baseline age and proportion of male participants were evaluated as continuous covariates. Furthermore, a meta‐regression was performed including study phase (OLE vs RCT) as a covariate. Meta‐regression results are reported as incidence rate ratios (IRRs) with 95% CrIs.

### 2.8 Risk‐of‐bias assessment

For the primary outcome, risk of bias (RoB) for RCTs was assessed independently by two reviewers (P.M. and M.S.B.) using the Cochrane RoB 2 tool. This tool is designed to evaluate the risk of bias across five key domains: (1) the randomization process, (2) deviations from intended interventions, (3) missing outcome data, (4) measurement of the outcome, and (5) selection of the reported result.^36^ For OLE studies, RoB was evaluated using the Risk Of Bias In Non‐randomized Studies of Interventions (ROBINS‐I) tool for non‐randomized studies of interventions, a tool designed to evaluate the RoB across seven key domains: (1) bias due to confounding, (2) bias in the selection of participants into the study, (3) bias in the classification of interventions, (4) bias due to deviations from intended interventions, (5) bias due to missing data, (6) bias in measurement of outcomes, and (7) bias in the selection of the reported result.^37^ Given that mortality and SUDEP are objective outcomes, particular attention was paid to completeness of follow‐up, outcome ascertainment, and potential misclassification. Discrepancies were resolved by consensus.

## 3 RESULTS

### 3.1 Search results and study characteristics

**FIGURE 1:**
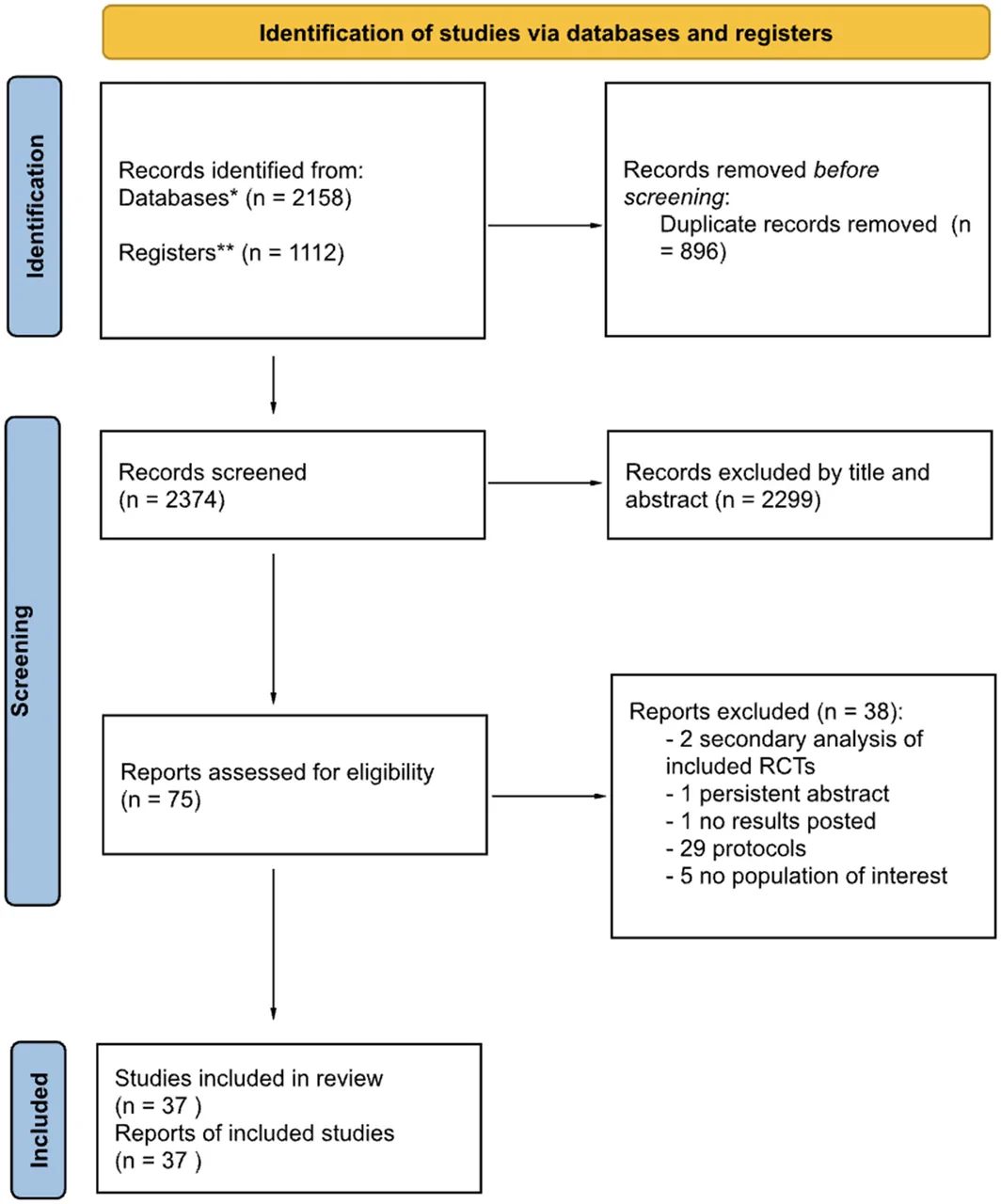
PRISMA 2020 flow diagram for new systematic reviews which included searches of databases and registers only. *PubMed, Cochrane; **ClinicalTrials.gov.

After screening 2374 non‐duplicated records, 37 studies met the inclusion criteria.^23^, ^26^, ^27^, ^29^, ^30^, ^38^, ^39^, ^40^, ^41^, ^42^, ^43^, ^44^, ^45^, ^46^, ^47^, ^48^, ^49^, ^50^, ^51^, ^52^, ^53^, ^54^, ^55^, ^56^, ^57^, ^58^, ^59^, ^60^, ^61^, ^62^, ^63^, ^64^, ^65^, ^66^, ^67^, ^68^, ^69^ Following full‐text assessment, 38 studies were excluded (Figure 1). Specifically, exclusions comprised two secondary analysis of included RCTs,^70^, ^71^ one persistent abstract,^72^ five studies that did not include the population of interest or lacked DEE‐specific data,^73^, ^74^, ^75^, ^76^, ^77^ one study with no results posted on ClinicalTrials.gov,^78^ and 29 study protocols corresponding to included RCTs.

All 37 eligible RCTs were included in the analysis, encompassing a total of 3757 patients with DEEs, including 998 with DS, 2221 with LGS, 369 with IESS, and 169 with other DEE syndromes, including cyclin‐dependent kinase‐like 5 (CDKL5) deficiency disorder (*n* = 105), DEE with spike–wave activation in sleep (DEE‐SWAS) (*n* = 24), KCNQ2‐DEE (*n* = 8), and glucose transporter 1 deficiency syndrome (*n* = 36). These were supplemented by 15 related long‐term OLE studies,^16^, ^17^, ^18^, ^19^, ^20^, ^21^, ^22^, ^23^, ^24^, ^25^, ^27^, ^28^, ^29^, ^31^, ^62^, ^68^ which included 2194 patients who continued follow‐up from 22 of the original RCTs. Characteristics of the RCT and OLE studies are summarized in Table 1.

### 3.2 Mortality and SUDEP outcomes

With pooling of the RCT and OLE studies, there were 37 deaths, including 2 definite SUDEP, 12 probable SUDEP, and 1 possible SUDEP case. Causes of death, including SUDEP classification, are detailed in Table 2.

**FIGURE 2:**
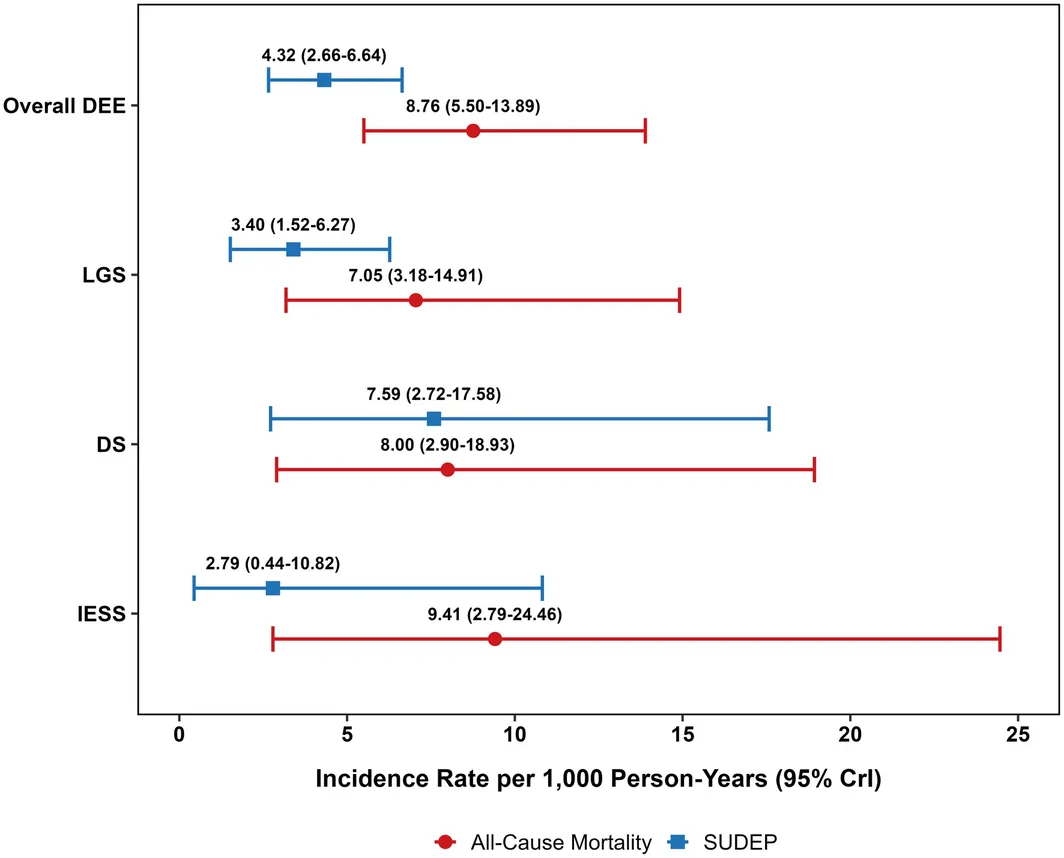
Incidence rates per 1000 person‐years for all‐cause mortality and sudden unexpected death in epilepsy (SUDEP) Points represent Bayesian estimates and horizontal bars indicate 95% credible intervals. DEE = developmental and epileptic encephalopathy; DS = Dravet Syndrome; IESS = Infantile Epileptic Spasm Syndrome; LGS = Lennox–Gastaut Syndrome.

In the overall DEE population, the Bayesian posterior median all‐cause mortality rate was 8.76 per 1000 PY (95% CrI: 5.50–13.89) (Figure 2). Sensitivity analysis using weakly informative priors yielded consistent, albeit slightly lower, estimates (see Data S1; eTable 2). The frequentist GLMM yielded a comparable estimate of 8.23 per 1000 PY (95% CI: 4.81–14.07). When stratifying the analysis by trial phase, the estimated mortality rate during RCTs was 8.91 deaths per 1000 PY (95% CrI: 4.68–16.57), whereas during OLEs it was estimated at 10.00 deaths per 1000 PY (95% CrI: 5.78–17.92).

The Bayesian posterior median SUDEP rate was 4.32 per 1000 PY (95% CrI: 2.66–6.64). Restricting the analysis to definite and probable cases yielded an estimate of 3.96 per 1000 PY (95% CrI: 2.43–6.18). Sensitivity analysis using weakly informative priors produced similar results (see Data S1; eTable 2). When stratifying the analysis by trial phase, the estimated SUDEP rate during RCTs was 4.78 deaths per 1000 PY (95% CrI: 2.41–8.72), whereas during OLEs it was estimated at 4.61 deaths per 1000 PY (95% CrI: 2.74–7.29).

### 3.3 Subgroup analysis

In the LGS subgroup (*n* = 2221) there were 27 deaths of which 8 were definite or probable SUDEP, and 1 was possible SUDEP. The Bayesian model estimated a posterior median all‐cause mortality rate of 7.05 per 1000 PY (95% CrI: 3.18–14.91). The Bayesian posterior median SUDEP rate was 3.4 per 1000 PY (95% CrI: 1.52–6.27), whereas restricting the analysis to definite and probable cases yielded an estimate of 2.84 per 1000 PY (95% CrI: 1.19–5.52).

In DS (*n* = 998), five deaths were reported, including one definite SUDEP plus, with the remaining cases classified as probable SUDEP. The Bayesian model estimated a posterior median all‐cause mortality rate of 8 per 1000 PY (95% CrI: 2.90–18.93), whereas the estimated posterior median probable/definite SUDEP rate was 7.59 per 1000 PY (95% CrI: 2.72–17.58).

In IESS (*n* = 369), there were four deaths and one probable SUDEP case. The Bayesian estimate for posterior median all‐cause mortality rate using weakly informative priors was 9.41 per 1000 PY (95% CrI: 2.79–24.46), whereas the SUDEP rate was 2.79 per 1000 PY (95% CrI: 0.44–10.82).

### 3.4 Meta‐regression analysis

Meta‐regression analysis did not identify any consistent associations between the examined covariates and outcomes. In the analysis assessing age, each 1‐year increase in mean cohort age was associated with an IRR of 0.94 (95% CrI: 0.84–1.04) for all‐cause mortality and 0.97 (95% CrI: 0.88–1.06) for SUDEP. Regarding sex distribution, each 1% increase in the proportion of males corresponded to an IRR of 0.97 (95% CrI: 0.91–1.03) for all‐cause mortality and 0.98 (95% CrI: 0.92–1.04) for SUDEP. Finally, the meta‐regression evaluating the study phase (with the RCT phase as the reference) yielded an IRR of 1.10 (95% CrI: 0.45–2.68) for all‐cause mortality and 0.86 (95% CrI: 0.36–2.13) for SUDEP.

### 3.5 Risk of bias assessment

RoB assessment using the Cochrane RoB 2 tool for the outcome of SUDEP indicated low to moderate risk of bias across the included RCTs. For OLE studies, the ROBINS‐I tool indicated an overall moderate to high RoB across studies (Data S1; eTables 3 and 4).

## 4 DISCUSSION

In this study, we provide, to our knowledge, the first estimates of all‐cause mortality and SUDEP incidence in DEEs derived from RCTs and long‐term OLE studies, thereby complementing prior evidence from retrospective cohorts and registries. We also report syndrome‐specific estimates, including the first systematic assessment of SUDEP in LGS.

Specifically, our Bayesian posterior estimate for all‐cause mortality in the overall DEE population was 8.76 per 1000 PY, whereas the SUDEP incidence was 4.32 per 1000 PY, with broadly consistent results across sensitivity analyses. SUDEP accounted for approximately half of all deaths, consistent with prior studies.^5^, ^6^, ^7^, ^8^, ^9^, ^10^, ^11^, ^35^ Direct comparisons with previous literature remain challenging because of differences in SUDEP definitions, case ascertainment, study design, population structure, and completeness of reporting.

Recent prospective evidence from a large multicenter epilepsy cohort by Ochoa‐Urrea et al. reported a high overall SUDEP incidence (definite, probable, and possible) of 4.76 per 1000 PY and a mortality rate of 12 per 1000 PY.^35^ Despite including only 17% of individuals with intellectual disability, and with the proportion of patients with DEEs not specified, the reported SUDEP incidence was higher than that observed in our analysis. This appears unexpected, as a higher SUDEP risk might be expected in DEEs compared with less severe epilepsies. Ochoa‐Urrea and colleagues reported several factors independently associated with increased SUDEP risk, including frequent generalized tonic–clonic seizures (GTCS), prolonged peri‐ictal central apnea, and living alone, the latter being a strong moderator of risk. In this setting, despite the severity of DEEs, differences in social and caregiving context, including greater awareness of mortality risk and closer supervision by caregivers in individuals with DEEs, may partly contribute to this discrepancy.^79^ By contrast, population‐based studies from Sweden, reported lower SUDEP estimates, with an incidence rate of 1.2 per 1000 PY, further underscoring the substantial variability of reported SUDEP incidence across study designs and populations.^80^

When focusing on the incidence rates of SUDEP and mortality observed in our meta‐analysis, these should be interpreted in light of at least three major limitations of RCT and OLE datasets, which could have potentially contributed to lower observed mortality and SUDEP estimates. First, most RCTs in DEEs are small and of limited duration. Although these designs are appropriate for evaluating short‐term ASM efficacy and common AEs, they are not designed to capture rare but clinically crucial outcomes such as all‐cause mortality or SUDEP. This issue is particularly relevant in DEEs, where mortality is uncommon within the timeframe of a typical RCT, yet remains a major determinant of long‐term prognosis.

Second, long‐term OLE studies, despite contributing additional person‐time, do not fully overcome this limitation. OLE cohorts are frequently affected by incomplete or inconsistent reporting of deaths, and in some studies, it remains unclear whether safety data refer to the entire originally enrolled population or exclude a subset of patients who did not complete the preplanned study duration. Moreover, the transition from RCT to OLE is inherently selective, as patients who complete the randomized phase and are more adherent to treatment are more likely to enter the extension. This introduces an important selection mechanism that may enrich OLE populations with individuals who are more stable, more treatment‐adherent, or otherwise less vulnerable than the source population.

Third, RCT populations may underrepresent the most clinically fragile DEE patients. Restrictive eligibility criteria, exclusion of severe comorbidities, recruitment in specialized centers, and the practical demands of trial participation may all result in the preferential inclusion of less vulnerable individuals. In addition, outcomes may be influenced by the trial environment itself. The intensive monitoring inherent to trial participation, including frequent visits, close supervision, and systematic AEs reporting, may itself reduce the likelihood of fatal outcomes compared with routine care. Taken together, these features may lead RCT‐based datasets to underestimate mortality and SUDEP compared with prospective observational cohorts and natural history studies. However, because our meta‐analysis was not designed as a direct comparative study and did not include an internal age‐matched non‐DEE control group, comparisons with external populations should be interpreted cautiously. Therefore, although trial‐related features concerning cohort structure, outcome ascertainment, and follow‐up may have contributed to lower observed mortality and SUDEP incidence, our study cannot determine whether, or to what extent, these rates underestimate the underlying mortality and SUDEP risk in DEEs.

In LGS, the estimated all‐cause mortality incidence was 7.05 per 1000 PY, lower than that reported in population‐based studies, which have described mortality rates of ~13 per 1000 PY, yet slightly higher than recent systematic reviews reporting 6.12 deaths per 1000 PY.^5^, ^6^ Notably, we provide, to our knowledge, the first systematic estimate of SUDEP incidence in this population, at 3.4 per 1000 PY. Although this estimate may be conservative in light of the above‐mentioned limitations, it indicates that SUDEP represents a substantial component of overall mortality in LGS.

In DS, the observed mortality rate was 8.0 per 1000 PY, slightly higher than in LGS and broadly aligned with the known high‐risk profile of this syndrome, but still lower than estimates from previous systematic reviews (15.84 deaths per 1000 PY).^6^ This difference is notable and likely reflects, at least in part, the same structural limitations discussed, including selection of less vulnerable patients into RCTs and OLEs, shorter observation windows, and differences in event ascertainment compared with registry‐based or natural history studies. Notably, all deaths observed in DS patients in our meta‐analysis were classified as probable or definite SUDEP, reinforcing the central clinical importance of SUDEP in this syndrome, where frequent drug‐resistant GTCS and *SCN1A*‐related autonomic and respiratory dysfunction may confer particularly high risk.^4^, ^5^, ^81^

In IESS, mortality estimates were characterized by substantial uncertainty because of the limited number of events and smaller sample size. The estimated all‐cause mortality rate was 9.41 per 1000 PY, the highest among the analyzed DEE subgroups, consistent with prior longitudinal studies reporting elevated mortality in IESS, often driven by underlying etiology.^8^, ^9^, ^10^, ^11^ SUDEP incidence was 2.79 per 1000 PY, in line with previous reports suggesting that a smaller proportion of deaths in IESS are attributable to SUDEP, possibly because GTCS, the most important risk factor for SUDEP, are usually absent in this patient population^11^; however, the wide credible intervals warrant cautious interpretation.

Notably, most included studies enrolled pediatric patients: 19 of 37 RCTs were conducted exclusively in children and adolescents, 17 included both pediatric and adult participants, and only 1 was restricted to adults. Previous estimates of SUDEP incidence in pediatric populations vary substantially according to epilepsy severity, etiology, and cohort characteristics, with higher incidence reported in selected genetic etiologies and in more severe epilepsy phenotypes.^82^ Although SUDEP has traditionally been considered more frequent among young adults, particularly those 20–40 years of age, recent population‐based studies specifically comparing incidence across age groups have suggested comparable rates in children and adults.^80^, ^83^ In DEEs, the impact of age on SUDEP and mortality remains insufficiently explored, although available evidence suggests that children with DEEs, particularly those with specific ion channelopathies, may be at high risk of SUDEP.^84^ In addition, systematic reviews and retrospective cohort studies indicate an early median age at death in several DEE syndromes. Donnan et al. reported a median age at SUDEP occurrence of 9.8 years in DEEs (range, 2.7–39 years), whereas Sullivan et al. reported a median age at death ranging from 4.7 to 7 years in DS cohorts, suggesting that mortality and SUDEP burden may occur early in life in patients with DEEs.^5^, ^6^ In the present study, meta‐regression did not identify a robust association between age and mortality or SUDEP. However, this finding should be interpreted cautiously, given the limited statistical power of study‐level meta‐regression and the possibility that median cohort age may reflect differences in syndrome composition and disease severity rather than an independent association between age and mortality or SUDEP risk.

Beyond the novel use of prospectively collected RCT and OLE data, an important strength of the present study is the application of a Bayesian hierarchical framework to model sparse data and rare events. Conventional frequentist approaches often perform poorly in this setting, particularly when many studies report zero events.^85^ By contrast, the Bayesian model allowed inclusion of all studies, incorporation of prior information, and generation of stable posterior estimates despite the rarity of outcomes. Consistency across Bayesian models, sensitivity analysis using weakly informative priors, and frequentist mixed‐effects models further supports the robustness of the statistical approach. Of note, this framework goes beyond a simple pooling of deaths over total person‐time, which would not account for between‐study heterogeneity or the dependence structure arising from linked RCT and OLE phases. By explicitly modeling these sources of variability, the hierarchical analysis provides more reliable incidence estimates, even if the underlying data remain subject to the design‐related limitations discussed.

By contrast, additional limitations should be acknowledged. First, the completeness of information on reported deaths was variable. Although we were able to classify most deaths across RCTs and OLE studies using publications and FDA review documents, detailed clinical circumstances were not consistently available. For a subset of deaths, only aggregate study‐level information was available, and classification of SUDEP relied on summary data reported by the original investigators. In addition, autopsy findings were rarely reported in probable SUDEP cases, thereby limiting the possibility of conducting sensitivity analysis according to different levels of SUDEP diagnostic certainty.^3^ Second, unlike previous meta‐analysis assessing the impact of ASM on risk of SUDEP,^86^ the rarity of deaths during the controlled phases of DEE trials precluded comparisons between the ASM and placebo arms. Likewise, the limited number of events precluded any comparisons among different ASMs and between the different monogenic causes of DEE included in the meta‐analysis, which could have provided useful information for SUDEP counseling. In this context, it is worth noting that, in a previous study by Cross and colleagues, the authors suggested a possible protective effect of fenfluramine on mortality and SUDEP by comparing the lower SUDEP and mortality rates observed in the RCT and OLE phases of fenfluramine studies with rates reported in the existing literature.^32^ Larger collaborative datasets, ideally integrating trial data with prospective disease‐specific registries and post‐marketing surveillance, will be required to determine whether treatment‐related differences in mortality or SUDEP can be reliably assessed in this population.

## 5 CONCLUSIONS

This Bayesian meta‐analysis provides the first estimates of all‐cause mortality and SUDEP in DEEs from RCTs and long‐term OLE studies, including the first systematic assessment of SUDEP in LGS. Limitations of trial design, small sample sizes, short follow‐up, selective OLE participation, incomplete reporting, and underrepresentation of vulnerable patients, should be considered when interpreting these findings, as they could have contributed to lower observed mortality and SUDEP estimates. SUDEP accounted for roughly half of all deaths in the overall DEE population and in LGS, with higher incidence in DS, highlighting its role as a major cause of death in these syndromes. These findings emphasize that standard efficacy trials alone may not fully capture mortality and SUDEP risk in patients with DEEs. Future studies with larger and more representative populations, with longer yet feasible follow‐up, are needed to better define the SUDEP risk in DEEs. Systematic death adjudication using standardized SUDEP criteria are recommended, ideally supported by autopsy data, when available, as well as a detailed structured reporting system. Finally, integration of real‐world data, including less selected and non‐tertiary care populations, is also essential to reduce selection bias and better inform long‐term management and trial design.

## AUTHOR CONTRIBUTIONS

P. Moro: drafting/revision of the manuscript for content, including medical writing for content; major role in the acquisition of data; and analysis or interpretation of data; M.S. Borioni: major role in the acquisition of data; drafting/revision of the manuscript for content, including medical writing for content; A. Mazzeo: major role in the acquisition of data; drafting/revision of the manuscript for content, including medical writing for content; C. Di Bonaventura: supervision; drafting/revision of the manuscript for content, including medical writing for content; E. Cocchi: drafting/revision of the manuscript for content, including medical writing for content; analysis or interpretation of data. E. Cerulli Irelli: drafting/revision of the manuscript for content, including medical writing for content; major role in the acquisition of data; study concept or design; analysis or interpretation of data.

## FUNDING INFORMATION

No targeted funding reported. This review was conducted without any specific financial or non‐financial support.

## CONFLICT OF INTEREST STATEMENT

P.M., E.C. and M.S.B. have no conflict of interests to disclose; A.M. has received speaker's fees from Angelini Pharma outside the submitted work; C.D.B. has received speaker's or consultancy fees from UCB Pharma, Eisai, GW Pharmaceuticals, JAZZ Pharmaceuticals, Angelini, Lusofarmaco, and Ecupharma outside the submitted work; E.C.I. has received speaker's fees from Angelini Pharma and travel support from UCB pharma, Jazz Pharmaceuticals and Angelini Pharma outside the submitted work.

## ETHICAL PUBLICATION STATEMENT

We confirm that we have read the Journal's position on issues involved in ethical publication and affirm that this report is consistent with those guidelines.

## Supporting information


Data S1.

## Data Availability

The data that support the findings of this study are available from the corresponding author upon reasonable request.
