# Supplementary material for: SUDEP and mortality in developmental and epileptic encephalopathies: A meta‐analysis of randomized clinical trials and extension studies
Source: Epilepsia. 2026 Jun 26;67(9):4631–48. doi: 10.1002/epi.70348 (PMC13592499; doi:10.1002/epi.70348)
Supplement: Supplementary file 1 [file EPI-67-4631-s001.docx]

**Supplementary methods**

Search Strategy

Pubmed

("Epileptic Encephalopathy" OR "Epileptic Encephalopathies" OR "Developmental and Epileptic

Encephalopathy" OR "Developmental and Epileptic Encephalopathies" OR DEE OR "Early

Infantile Epileptic Encephalopathy" OR EIEE OR "Ohtahara syndrome" OR "Infantile Spasms" OR

"West syndrome" OR "Dravet syndrome" OR "Severe Myoclonic Epilepsy of Infancy" OR SMEI

OR "Epilepsy of Infancy with Migrating Focal Seizures" OR "Lennox-Gastaut Syndrome"[Mesh]

OR "Lennox-Gastaut syndrome" OR LGS OR "Myoclonic-Atonic Epilepsy" OR "Doose

syndrome" OR "Epileptic Encephalopathy with Continuous Spike-and-Wave during Sleep" OR

CSWS OR ESES OR "Tuberous Sclerosis"[Mesh] OR "Tuberous Sclerosis" OR KCNQ2 OR

KCNT1 OR SCN1A OR SCN2A OR SCN8A OR STXBP1 OR CDKL5 OR TSC1 OR TSC2 OR

GLUT1 OR SLC2A1 ) AND ( "Anticonvulsants"[Mesh] OR "anti-seizure medication" OR

"antiseizure medication" OR "anti-epileptic drug" OR "anti-seizure drug" OR ASM OR AED OR

Acetazolamide OR Ataluren OR Bexicaserin OR Brivaracetam OR Bromide OR "Potassium

bromide" OR Cannabidiol OR Carbamazepine OR Cenobamate OR Clobazam OR Clonazepam OR

Clorazepate OR Diazepam OR Divalproex OR Eslicarbazepine OR Ethosuximide OR Felbamate

OR Fenfluramine OR Fosphenytoin OR Gabapentin OR Ganaxolone OR Lacosamide OR

Lamotrigine OR Levetiracetam OR Lorazepam OR Losigamone OR Midazolam OR Nitrazepam

OR Oxcarbazepine OR Padsevonil OR Paraldehyde OR Perampanel OR Phenobarbital OR

Phenytoin OR Pregabalin OR Primidone OR Retigabine OR Rufinamide OR Stiripentol OR

Sulthiame OR Sultiame OR Tiagabine OR Topiramate OR Valproate OR "Valproic acid"[Mesh]

OR Vigabatrin OR Zonisamide ) AND (randomized controlled trial[pt] OR controlled clinical

trial[pt] OR randomized[tiab] OR placebo[tiab] OR "randomly allocated"[tiab] OR trial[tiab] OR

groups[tiab])

Cochrane

(“Epileptic Encephalopathy” OR “Epileptic Encephalopathies” OR “Developmental and Epileptic

Encephalopathy” OR “Developmental and Epileptic Encephalopathies” OR DEE OR “Early

Infantile Epileptic Encephalopathy” OR EIEE OR “Ohtahara syndrome” OR “Infantile Spasms”

OR “West syndrome” OR “Dravet syndrome” OR “Severe Myoclonic Epilepsy of Infancy” OR

SMEI OR “Epilepsy of Infancy with Migrating Focal Seizures” OR “Lennox-Gastaut Syndrome”

OR LGS OR “Myoclonic-Atonic Epilepsy” OR “Doose syndrome” OR “Epileptic Encephalopathy

with Continuous Spike-and-Wave during Sleep” OR CSWS OR ESES OR “Tuberous Sclerosis”

OR KCNQ2 OR KCNT1 OR SCN1A OR SCN2A OR SCN8A OR STXBP1 OR CDKL5 OR TSC1

OR TSC2 OR GLUT1 OR SLC2A1) AND (“Anti-seizure medication” OR “antiseizure medication”

OR “anti-epileptic drug” OR “anti-seizure drug” OR ASM OR AED OR Acetazolamide OR

Ataluren OR Bexicaserin OR Brivaracetam OR Bromide OR Potassium bromide OR Cannabidiol

OR Carbamazepine OR Cenobamate OR Clobazam OR Clonazepam OR Clorazepate OR

Diazepam OR Divalproex OR Eslicarbazepine OR Ethosuximide OR Felbamate OR Fenfluramine

OR Fosphenytoin OR Gabapentin OR Ganaxolone OR Lacosamide OR Lamotrigine OR

Levetiracetam OR Lorazepam OR Losigamone OR Midazolam OR Nitrazepam OR Oxcarbazepine

OR Padsevonil OR Paraldehyde OR Perampanel OR Phenobarbital OR Phenytoin OR Pregabalin

OR Primidone OR Retigabine OR Rufinamide OR Stiripentol OR Sulthiame OR Sultiame OR

Tiagabine OR Topiramate OR Valproate OR Valproic acid OR Vigabatrin OR Zonisamide)

Clinicaltrials.gov

(Advanced Search: Study Type - Interventional; Study Results - With results; Study Phase -

Phase 1, Phase 2, Phase 3, Phase 4)

Epileptic Encephalopathy OR Epileptic Encephalopathies OR Developmental and Epileptic

Encephalopathy OR Developmental and Epileptic Encephalopathies OR DEE OR Early Infantile

Epileptic Encephalopathy OR EIEE OR Ohtahara syndrome OR Infantile Spasms OR West

syndrome OR Dravet syndrome OR Severe Myoclonic Epilepsy of Infancy OR SMEI OR Epilepsy

of Infancy with Migrating Focal Seizures OR Lennox-Gastaut Syndrome OR LGS OR Myoclonic-

Atonic Epilepsy OR Doose syndrome OR Epileptic Encephalopathy with Continuous Spike-and-

Wave during Sleep OR CSWS OR ESES OR Tuberous Sclerosis OR KCNQ2 OR KCNT1 OR

SCN1A OR SCN2A OR SCN8A OR STXBP1 OR CDKL5 OR TSC1 OR TSC2 OR GLUT1 OR

SLC2A1 AND Anti-seizure medication OR antiseizure medication OR anti-epileptic drug OR antiseizure

drug OR ASM OR AED OR Acetazolamide OR Ataluren OR Bexicaserin OR Brivaracetam

OR Bromide OR Potassium bromide OR Cannabidiol OR Carbamazepine OR Cenobamate OR

Clobazam OR Clonazepam OR Clorazepate OR Diazepam OR Divalproex OR Eslicarbazepine OR

Ethosuximide OR Felbamate OR Fenfluramine OR Fosphenytoin OR Gabapentin OR Ganaxolone

OR Lacosamide OR Lamotrigine OR Levetiracetam OR Lorazepam OR Losigamone OR

Midazolam OR Nitrazepam OR Oxcarbazepine OR Padsevonil OR Paraldehyde OR Perampanel

OR Phenobarbital OR Phenytoin OR Pregabalin OR Primidone OR Retigabine OR Rufinamide OR

Stiripentol OR Sulthiame OR Sultiame OR Tiagabine OR Topiramate OR Valproate OR Valproic

acid OR Vigabatrin OR Zonisamide

**Statistical Analysis**

*Bayesian Model Specification*

Incidence rates were estimated using a Bayesian hierarchical generalized linear mixed model (GLMM). To account for possible overdispersion, overall mortality was modeled using a negative binomial likelihood. In contrast, SUDEP was modeled using a Poisson likelihood, as the extreme rarity of events and posterior predictive checks did not indicate overdispersion. Between-study variability was modeled using a random intercept at the drug development program level, rather than at the individual trial phase level, to account for between-program heterogeneity and within-program correlation. This hierarchical structure prevents pseudoreplication, as OLEs largely include rollover participants from the initial RCTs; treating phases as independent would violate assumptions of independence and overstate precision. In addition, rare outcomes such as SUDEP depend strongly on cumulative exposure time. Clustering at the program level allows information to be shared across RCT and OLE phases within the same program, improving stability of estimates and reducing the influence of short-duration RCTs, which often observe zero events, on overall incidence estimates. Person-years (PY) of follow-up were incorporated as an offset term to model incidence rates rather than raw event counts.

Given the rarity of events and sparse counts, moderately informative priors were specified for the overall log-incidence rates. Between–study-phase heterogeneity was modeled using a half-normal prior with scale 0.5 on the log-rate scale, allowing moderate heterogeneity (approximately ±65% multiplicative variation per standard deviation) while limiting implausibly large dispersion.

*Prior Elicitation and Baseline Selection*

Because the pooled cohort was primarily composed of patients with LGS, DS, and ISS, prior means were informed mainly by mortality and SUDEP data reported for these syndromes. For all-cause mortality, baseline rates for each syndrome were derived from previous population-based studies and long-term observational studies. Reported mortality estimates were 13.92 deaths per 1,000 PY (95% CI 7.19–24.31) for LGS, 15.8 deaths per 1,000 PY (95% CI 9.01–27.85) for DS, and 12.5 deaths per 1,000 PY (95% CI 10.28–15.06) for ISS.^1-6^

Additionally, a recent prospective multicenter study in individuals with epilepsy reported a mortality rate of 96 deaths among 7,982 individuals, corresponding to 12.03 deaths per 1,000 PY (95% CI 9.74–14.69).^7^ Considering the higher expected mortality rate in DEE populations,^7^ a baseline mortality of approximately 14 deaths per 1,000 PY was selected, corresponding to a log-scale prior of Normal(−4.27, 0.5). This prior allows substantial uncertainty, corresponding approximately to a range of 5.2 to 37.5 deaths per 1,000 PY on the incidence scale.

For SUDEP, evidence from a previous study of genetic DEEs indicates that SUDEP accounts for approximately 48% of all-cause mortality in these populations,^8^ with a similar proportion reported in individuals with epilepsy (include CI).^7^ Applying this proportional relationship resulted in an expected SUDEP incidence of approximately 7 deaths per 1,000 PY, corresponding to a log-scale prior of Normal(−4.96, 0.5). This prior allows substantial uncertainty, ranging from approximately 2.6 to 18.7 deaths per 1,000 PY.

*Subgroup-Specific Priors*

For syndrome-specific subgroup analysis, priors were anchored to syndrome-specific mortality baselines while applying the same proportional rule when direct SUDEP estimates were limited. Given the smaller sample sizes expected in these subgroup analysis, weakly informative priors were used to stabilize the models while allowing the estimates to be primarily driven by the observed data. The same Bayesian hierarchical GLMM was used. A negative binomial likelihood was used for Lennox–Gastaut syndrome mortality to account for overdispersion, whereas Poisson likelihoods were used for Dravet syndrome and infantile epileptic spasms syndrome mortality, as well as for SUDEP outcomes, given the rarity of events and lack of evidence of overdispersion.

For all-cause mortality, the following priors were specified according to the previously mentioned studies:^1-6^

- DS: Normal(−4.15, 1.5), with uncertainty ranging from 0.83 to 298.20 deaths per 1,000 PY.
- LGS: Normal(−4.27, 1.5), with uncertainty ranging from 0.74 to 264.47 deaths per 1,000 PY.
- ISS: Normal(−4.38, 1.5), with uncertainty ranging from 0.66 to 236.93 deaths per 1,000 PY.

For SUDEP, the log-incidence priors were:

- DS: Normal(−4.7, 1.5), with a prior SUDEP estimate centered at 9.1 deaths per 1,000 PY and uncertainty ranging from 0.48 to 172.04 deaths per 1,000 PY.
- LGS: Normal(−5.04, 1.5), with a prior estimate centered at 6.5 deaths per 1,000 PY, ranging from 0.34 to 122.46 deaths per 1,000 PY.
- ISS: Normal(−5.12, 1.5), with a prior estimate centered at 6 deaths per 1,000 PY, ranging from 0.32 to 113.04 deaths per 1,000 PY.

*Sensitivity Analysis*

To evaluate the influence of prior assumptions, comprehensive sensitivity analysis were conducted on both the baseline intercept and the between-study heterogeneity parameters. For the baseline intercept, we tested weakly informative priors with wider dispersion (SD = 1.5 on the log scale). On the incidence scale, this permitted broad prior ranges (i.e., 0.74 to 264.5 deaths per 1,000 PY for all-cause mortality), ensuring that posterior estimates were primarily driven by the observed trial data rather than prior constraints. We additionally evaluated an essentially flat baseline prior (SD = 10.0).

Additionally, estimates were compared with those obtained from a frequentist Poisson GLMM to evaluate consistency between Bayesian and frequentist approaches, employing the same random effect structure used in the Bayesian model. A further sensitivity analysis restricted to RCT and OLE phases only was performed to evaluate mortality and SUDEP estimates independently in the two phases.

*Model Fitting and Convergence Assessment*

Models were fitted using the *brms* package in the R programming language. Four Markov chains were run with 4,000 iterations each, including 2,000 warm-up iterations. Convergence was assessed using the Gelman–Rubin diagnostic (R̂ < 1.01) and visual inspection of trace plots.

Posterior incidence rates per 1,000 PY were obtained by exponentiating the posterior draws of the model intercept and scaling the resulting rates to 1,000 PY. Point estimates were reported as posterior medians of the transformed rate distribution, with 95% credible intervals (CrIs) defined by the 2.5th and 97.5th percentiles.

**References**

1. Autry AR, Trevathan E, Van Naarden Braun K, Yeargin-Allsopp M. Increased risk of death among children with Lennox-Gastaut syndrome and infantile spasms. *J Child Neurol*. 2010;25(4):441-447. doi:10.1177/0883073809348355

2. Cooper MS, Mcintosh A, Crompton DE, et al. Mortality in Dravet syndrome. *Epilepsy Res*. 2016;128:43-47. doi:10.1016/j.eplepsyres.2016.10.006

3. Sullivan J, Benítez A, Roth J, et al. A systematic literature review on the global epidemiology of Dravet syndrome and Lennox-Gastaut syndrome: Prevalence, incidence, diagnosis, and mortality. *Epilepsia*. 2024;65(5):1240-1263. doi:10.1111/epi.17866

4. Riikonen R. Long-term outcome of patients with West syndrome. *Brain Dev*. 2001;23(7):683-687. doi:10.1016/s0387-7604(01)00307-2

5. Harini C, Nagarajan E, Bergin AM, et al. Mortality in infantile spasms: A hospital-based study. *Epilepsia*. 2020;61(4):702-713. doi:10.1111/epi.16468

6. Iype M, Anish TS, Saradakutty G, Kunju PM, Sreedharan M, Ahamed SM. Long-term survival and factors associated with mortality among children with infantile epileptic spasms syndrome - A retrospective cohort study. *Seizure*. 2023;112:18-25. doi:10.1016/j.seizure.2023.09.005

7. Ochoa-Urrea M, Luo X, Vilella L, et al. Risk markers for sudden unexpected death in epilepsy: an observational, prospective, multicentre cohort study. *Lancet*. 2025;406(10511):1497-1507. doi:10.1016/S0140-6736(25)01636-8

8. Donnan AM, Schneider AL, Russ-Hall S, Churilov L, Scheffer IE. Rates of Status Epilepticus and Sudden Unexplained Death in Epilepsy in People With Genetic Developmental and Epileptic Encephalopathies. *Neurology*. 2023;100(16):e1712-e1722. doi:10.1212/WNL.0000000000207080

**eTable 1. PRISMA 2020 check-list**

| **eTable 2. Sensitivity analysis of the Bayesian models using alternative priors for the baseline log-incidence** | | |
| --- | --- | --- |
| Prior specification (baseline intercept) | Incidence rate (per 1,000 PY) | 95% Credible Interval |
| Mortality | | |
| Weakly informative (SD = 1.5) | 7.8 | 4.44-12.82 |
| Flat prior (SD = 10) | 7.63 | 4.35-12.92 |
| SUDEP | | |
| Weakly informative (SD = 1.5) | 3.82 | 2.1-6.26 |
| Flat prior (SD = 10) | 3.74 | 2-6.14 |

**eTable 3. Risk of bias summary for randomized studies (RoB 2) for the outcome of SUDEP**

| **Study** | **Bias from randomization process** | **Bias due to deviations from intended interventions** | **Bias due to missing outcome data** | **Bias in measurement of the outcomes** | **Bias in selection of the reported result** | **Overall risk of bias** |
| --- | --- | --- | --- | --- | --- | --- |
| Devinsky, 2021 | Some concerns | Some concerns | Some concerns | Some concerns | Low risk | Some concerns |
| DLugos, 2025 | Low risk | Low risk | Some concerns | Some concerns | Low risk | Some concerns |
| Devinsky, 2018  (GWPCARE1a) | Low risk | Low risk | Some concerns | Some concerns | Low risk | Some concerns |
| Devinsky, 2017  (GWPCARE1b) | Low risk | Low risk | Some concerns | Some concerns | Low risk | Some concerns |
| Miller, 2020  (GWPCARE2) | Low risk | Low risk | Some concerns | Some concerns | Low risk | Some concerns |
| Devinsky, 2018  (GWPCARE3) | Low risk | Low risk | Some concerns | Some concerns | Low risk | Some concerns |
| Thiele, 2018  (GWPCARE4) | Low risk | Some concerns | Some concerns | Some concerns | Low risk | Some concerns |
| Ng,2011 | Low risk | Some concerns | Some concerns | Some concerns | Low risk | Some concerns |
| NCT00162981,  2012 | Low risk | Low risk | Some concerns | Some concerns | Low risk | Some concerns |
| NCT04639310, 2020 | Low risk | Low risk | Some concerns | Some concerns | Some concerns | Some concerns |
| Ritter, 1993 | Low risk | Low risk | Some concerns | Some concerns | Low risk | Some concerns |
| Siegel, 1999 | Low risk | Low risk | Some concerns | Some concerns | Low risk | Some concerns |
| Nabbout, 2019 | Low risk | Low risk | Some concerns | Some concerns | Low risk | Some concerns |
| Sullivan, 2023 | Low risk | Low risk | Low risk | Low risk | Low risk | Low risk |
| Lagae, 2019 | Low risk | Low risk | Some concerns | Some concerns | Low risk | Some concerns |
| Knupp, 2022 | Low risk | Low risk | Low risk | Low risk | Low risk | Low risk |
| Knight, 2022 | Low risk | Low risk | Some concerns | Some concerns | Low risk | Some concerns |
| Sullivan, 2023 | Low risk | Low risk | Some concerns | Some concerns | Low risk | Some concerns |
| NCT00441896, 2007 | Low risk | Low risk | Some concerns | Some concerns | Some concerns | Some concerns |
| Motte, 1997 | Low risk | Low risk | Some concerns | Some concerns | Low risk | Some concerns |
| NCT04625101, 2020 | Low risk | Low risk | Some concerns | Some concerns | Some concerns | Some concerns |
| Vossler, 2024 | Low risk | Low risk | Some concerns | Some concerns | Low risk | Some concerns |
| Arzimanoglou, 2019 | Low risk | Low risk | Some concerns | Some concerns | Low risk | Some concerns |
| Glauser, 2008 | Low risk | Low risk | Some concerns | Some concerns | Low risk | Some concerns |
| Ohtsuka, 2014 | Low risk | Low risk | Some concerns | Some concerns | Low risk | Some concerns |
| Hahn, 2022 | Low risk | Low risk | Some concerns | Some concerns | Low risk | Some concerns |
| Halford, 2021 | Low risk | Low risk | Some concerns | Some concerns | Low risk | Some concerns |
| NCT04938427, 2021 | Low risk | Low risk | Some concerns | Some concerns | Some concerns | Some concerns |
| NCT04940624, 2021 | Low risk | Low risk | Some concerns | Some concerns | Some concerns | Some concerns |
| Chiron, 2000 | Low risk | Low risk | Some concerns | Some concerns | Low risk | Some concerns |
| Guerrini, 2002 | **NA** | **NA** | **NA** | **NA** | **NA** | **NA** |
| Debus, 2004 | Low risk | Low risk | Some concerns | Some concerns | Low risk | Some concerns |
| Sachdeo, 1999 | Low risk | Low risk | Some concerns | Some concerns | Low risk | Some concerns |
| Striano, 2022 | Low risk | Low risk | Some concerns | Some concerns | Low risk | Some concerns |
| Appleton, 1999 | Low risk | Low risk | Some concerns | Some concerns | Low risk | Some concerns |
| Elterman, 2001 | Low risk | Low risk | Low risk | Low risk | Low risk | Low risk |
| Dyken, 1985 | Low risk | Low risk | Some concerns | Some concerns | Low risk | Some concerns |

**eTable 4. Risk of bias summary for non-randomized studies (ROBINS-I) for the outcome of SUDEP**

| **OLE study** | **Bias due to confounding** | **Bias in selection of participants** | **Bias in classification of interventions** | **Bias due to deviations from intended interventions** | **Bias due to missing data** | **Bias in measurement of outcomes** | **Bias in selection of the reported result** | **Overall risk of bias judgement** |
| --- | --- | --- | --- | --- | --- | --- | --- | --- |
| Devinsky, 2019 | Moderate | Low risk | Low risk | Moderate | High risk | High risk | Low risk | High risk |
| Patel, 2021 | Moderate | Low risk | Low risk | Moderate | High risk | High risk | Low risk | High risk |
| Conry, 2014 | Moderate | Low risk | Low risk | Moderate | High risk | Moderate | Low risk | High risk |
| Dodson, 1993 | Moderate | Low risk | Low risk | Moderate | High risk | High risk | Low risk | High risk |
| Sullivan, 2020 | Moderate | Low risk | Low risk | Moderate | Moderate | Moderate | Low risk | Moderate |
| Knupp, 2022 | Moderate | Low risk | Low risk | Moderate | High risk | Moderate | Low risk | High risk |
| Olson, 2023 | Moderate | Low risk | Low risk | Moderate | High risk | High risk | Low risk | High risk |
| Vossler, 2024 | Moderate | Low risk | Low risk | Moderate | High risk | High risk | Low risk | High risk |
| Kluger, 2010 | Moderate | Low risk | Low risk | Moderate | High risk | High risk | Low risk | High risk |
| Ohtsuka, 2016 | Moderate | Low risk | Low risk | Moderate | High risk | High risk | Low risk | High risk |
| Halford, 2021 | Moderate | Low risk | Low risk | Moderate | High risk | High risk | Low risk | High risk |
| Debus, 2004 | Moderate | Low risk | Low risk | Moderate | High risk | High risk | Low risk | High risk |
| Glauser, 2000 | Moderate | Low risk | Low risk | Moderate | High risk | High risk | Low risk | High risk |
| Striano, 2022 | Moderate | Low risk | Low risk | Moderate | High risk | High risk | Low risk | High risk |
| Appleton, 1999 | Moderate | Low risk | Low risk | Moderate | High risk | Low risk | Low risk | High risk |
| Elterman, 2010 | Moderate | Low risk | Low risk | Moderate | High risk | Moderate | Low risk | High risk |
